# Altered Complement System Activity in Schizophrenia: Overexpression of C4 and/or Abnormal Expression of Complement Control Proteins in the DLPFC, Parietal Cortex, Temporal Cortex, Associative Striatum, Hippocampus, Cerebellum and Whole Blood

**DOI:** 10.1192/j.eurpsy.2022.930

**Published:** 2022-09-01

**Authors:** R. Rey, M. Leboyer

**Affiliations:** 1 INSERM, U1028; CNRS, UMR5292; Lyon Neuroscience Research Center, Psychiatric Disorders: from Resistance to Response Team, Lyon, F-69000, France, Schizophrenia Expert Center Of Lyon, Bron, France; 2 APHP CHU Mondor, Psychiatrie, Créteil, France

**Keywords:** Complement system, schizophrénia, Brain, Gene expression

## Abstract

**Introduction:**

In schizophrenia, abnormal synaptic pruning during adolescence may be due to an altered Complement system activity. While this hypothesis is supported by *C4* overexpression in various brain regions of individuals with schizophrenia, such alterations should be replicated and extended to other brain regions relevant to schizophrenia. Moreover, transcriptional studies of genes coding for proteins regulating the Complement system activity are lacking. Furthermore, it remains unknown whether cerebral and peripheral expression of *C4* and Complement control proteins (CCP) are related.

**Objectives:**

To identify altered expression of *C4* and CCP (*CSMD1*, *CSMD2*, *CD46*) coding genes at the cerebral and peripheral levels in schizophrenic individuals.

**Methods:**

We explored *C4* and CCP coding genes expression at the cerebral and peripheral levels. Using *shiny*GEO application we analyzed gene expression from eight Gene Expression Omnibus datasets obtained from 196 schizophrenic individuals and 182 control subjects. First, we compared gene expression between schizophrenic patients and controls in postmortem cerebral samples from 7 different brain regions. Then, we compared gene expression between schizophrenic patients and controls in 4 peripheral tissues.

**Results:**

We observed *C4* overexpression in the DLPFC, parietal, temporal cortex and associative striatum of schizophrenic individuals. We report altered transcriptional patterns of CCP genes in the DLPFC, hippocampus and cerebellum of schizophrenic individuals. *CD46* expression was altered in opposite directions between brain and blood of schizophrenic individuals. No significant alteration of *C4* expression was observed in peripheral tissues.

**Conclusions:**

Our results support the hypothesis of an altered Complement system activity in various brain regions of schizophrenic individuals which may disrupt the synaptic pruning process during adolescence.

**Disclosure:**

No significant relationships.

